# Reliability and Validity of the Modified Dental Anxiety Scale Among Children Aged 9 to 12 Years

**DOI:** 10.3390/dj13060248

**Published:** 2025-05-30

**Authors:** Satu Lahti, Mika Kajita, Vesa Pohjola, Auli Suominen

**Affiliations:** 1 Department of Community Dentistry, University of Turku, 20014 Turku, Finland; mika.ogawa@utu.fi (M.K.); vesa.pohjola@utu.fi (V.P.);; 2 Systemic Approaches to Improve Cardiometabolic and Brain Health during Lifespan (SYS-LIFE) Program Co-Founded by University of Turku and European Union, Kiinamyllynkatu 10, 20520 Turku, Finland; 3 Research Unit of Population Health, Faculty of Medicine, University of Oulu, 90014 Oulu, Finland

**Keywords:** dental fear, dental anxiety, children, reliability, validity

## Abstract

**Objectives:** Our aim was to study whether the Modified Dental Anxiety Scale (MDAS) is reliable and valid for use in children aged 9 to 12 years. **Methods:** The study population was a convenient sample of Finnish comprehensive school pupils in the third and sixth grades (N = 57 and N = 69, respectively). Dental fear and anxiety (DFA) were measured with the Finnish validated adult version of MDAS, the modified Child Fear Survey Schedule—Dental Subscale (CFSS-DS-M), and a single question. Reliability was evaluated using Cronbach’s alpha. The criterion validity of MDAS was assessed using Spearman rank correlation coefficients against CFSS-DS-M and the single question. Construct validity was assessed by examining the ability of MDAS to find differences according to gender and treatment procedures using the chi-square test for categorized and the Mann–Whitney and Jonckheere–Terpstra tests for continuous variables. **Results:** The Cronbach alphas were 0.841, 0.708, and 0.778 for MDAS total, anticipatory, and treatment-related DFA, respectively. Correlations between MDAS and CFSS-DS-M total and subscale scores were moderate to strong (ρ = 0.559–0.794), supporting criterion validity. Girls in third grade had lower mean MDAS anticipatory DFA (3.4, SD = 1.44) than boys (4.5, SD = 2.21, *p* = 0.051). In sixth graders, girls had higher mean MDAS treatment-related DFA (8.4, SD = 3.17) than boys (6.9, SD = 2.61, *p* = 0.067). Children reporting orthodontic treatment had lower anticipatory DFA (mean = 3.4, SD = 2.13) than children not reporting (mean = 4.0, SD = 1.83; *p* = 0.009), supporting construct validity. **Conclusions:** The Finnish version of the MDAS showed good reliability, good criterion validity, and acceptable construct validity, supporting its use in children aged 9–12 years.

## 1. Introduction

Dental fear and anxiety (DFA) are common in children and adolescents. The global pooled prevalences reported in a systematic review and meta-analysis were 37% for preschoolers, 26% for schoolchildren, and 13% for adolescents [1]. Among 2- to 6-year-olds, the pooled prevalence was 30% [2]. DFA is related to poorer oral health [3,4] and poorer oral health-related quality of life and self-esteem [5]. Thus, if not assessed and treated, DFA can have severe consequences for children.

Several measures have, according to a systematic review, been developed to assess the DFA of children [6]. While many DFA measures were found reliable and valid, this systematic review noted that most assessed only selected aspects of DFA without a comprehensive framework. A more recent systematic review on measures of DFA and stress revealed that the Child Fear Survey Schedule—Dental Subscale (CFSS-DS) was the most used [7], as it was in another review [6]. Several other measures have also been used. Of the non-projective multi-item measures, most include only items directly related to oral healthcare treatment procedures [6,7]. Of the reviewed measures, the Dental Anxiety Scale (DAS), the Modified Dental Anxiety Scale (MDAS), the short version of the Dental Anxiety Inventory (S-DAI), Smiley Faces Program Revised (SFP-R), and the Dental Fear Survey (DFS) include items that measure anticipatory element of DFA [8,9,10,11,12]. The CFSS-DS and the Modified Child Dental Anxiety Scale (MCDAS) include a general item assessing how afraid the child is of the dentist or of going to the dentist, respectively [6,13], which may assess the anticipatory element. However, most of these measures did not consider the anticipatory element of DFA separately, although it is theoretically relevant, in addition to the situational aspects of DFA [14]. Assessing anticipatory DFA is important, as it might capture other sources of DFA than own treatment procedures [15]. It might also help in identifying those children at risk of avoiding oral healthcare.

The most often used measure, CFSS-DS, usually contains 15 items, but the number of items and factors obtained have also varied across studies [16,17,18,19,20]. Also, the informant (child vs. parent) and the answering scheme have varied [17,19,20]. MDAS contains only five items, and it has been widely used with different language versions tested for reliability and validity [21] and has an established cut-off [22]. Though MDAS was developed for use in adults [9], it could be useful also for children due to its brevity. A review on children as respondents in surveys noted that though the cognitive development of children allows them to be surveyed with questionnaires generally from age 8, the measures should be short, as younger children lack concentration and motivation [23].

A meta-analysis showed that parental and child DFA are associated [24], particularly among children aged 8 years or younger. However, the strength of this association seemed to be affected by the assessment methods used. In addition, parents and children at older ages could not assess each other’s DFA [25,26]. Thus, there is a need for child-report measures that are short but also allow for comparison within family members. The authors of the meta-analysis suggested that state-of-the-art scales should be used to assess dental fear in parents and children [24]. As MDAS is widely used in adults, its adaptation for school-aged children could allow for its use in larger surveys, in oral healthcare settings, and in comparisons across age groups, for example, with siblings and with parents in future research. Thus, our aim was to study whether MDAS is reliable and valid for use in children aged 9 to 12 years.

## 2. Materials and Methods

The study population was a convenient sample of Finnish pupils in the third and sixth grades (N = 67 and N = 74, respectively) attending comprehensive school on the day of study. Teachers of the respective classes administered the paper-format questionnaires to the children during the school day. The parents of the children gave written informed consent. The study protocol was approved by the Research Permit Section of the Strategy and Development Unit of the City of Tampere, Finland (17 February 2021). The sample size for a cross-over study with two sequences was calculated using R (package *trialsize* version 1.4, function TwoSampleCrossOver. Equivalence). The effect size margin of 2 was derived from the true mean difference (mean for Child Fear Survey Schedule—Dental Subscale, modified version CFSS-DS-M = 37 and standardized mean for MDAS = 35) and the standard deviation of 10. The non-inferiority margin was set at 0.1. With a power of 80% (beta = 0.2) and an alpha level of 0.05, the sample size was estimated to be 85 subjects in both equal-sized sequences. For a power of 90%, the sample size was estimated to be 118 subjects.

A Finnish version of MDAS, which has been found valid and reliable in adults, was used [27]. The validated Finnish scale to measure child DFA, i.e., CFSS-DS-M (11-item version modified from the original 15-item version), was used for assessing criterion validity [17]. In addition, a single question, namely “How afraid of a dentist are you?”, was used. In half of the questionnaires, the order of the measures was MDAS before CFSS-DS-M and in the other CFSS-DS-M before MDAS for the other half. The questionnaires with different orders of dental fear measures were randomly assigned to participants to avoid bias from the order of the measures.

MDAS measured DFA using five questions: (1) If you went to your dentist for treatment tomorrow, how would you feel? (2) If you were sitting in the waiting room (waiting for treatment), how would you feel? (3) If you were about to have a tooth drilled, how would you feel? (4) If you were about to have your teeth scaled and polished, how would you feel? (5) If you were about to have a local anesthetic injection in your gum above an upper back tooth, how would you feel? The five-point response alternatives ranged from 1 (not anxious) to 5 (extremely anxious) [9].

CFSS-DS-M measured DFA in 11 situations: in general, when keeping the mouth open, at the dentist, teeth being cleaned by a dentist or an assistant, drilling, local anesthesia, hearing the sound of drilling, being unable to breathe, instruments put in the mouth, suction used in the mouth, and dental treatment causing pain. The questions used 5-point Likert-scale response alternatives from 1 (not afraid) to 5 (very afraid) and an alternative, 6 (no experience of this particular matter). The single question “How afraid are you of dental treatment in general?” used the same response alternatives as for CFSS-DS-M [17].

For assessing construct validity, children were also asked for their gender, age in years, and whether they had ever received fillings, extractions, local anesthesia, or orthodontic treatment, with response options of yes or no. A variable number of treatment categories (range 0 to 4) was calculated by summing up categories from which the child had experiences. One participant did not report gender and was excluded from analyses.

For MDAS, sum scores for all items (range = 5–25) and two subscales, that is, anticipatory dental anxiety (ADA) (items 1 and 2; score range = 2−10) and treatment-related dental (TRA) anxiety (items 3, 4, and 5; score range = 3−15), were calculated [14]. One participant reported missing values for MDAS items 1, 2, and 5, and these missing values were not imputed. One participant reported two alternatives for MDAS item 1, and this was treated as missing.

For CFSS-DS-M, a total sum and sum for two subscales were calculated [17]. Of the subscales, attending dentist (AD) included the following items: fear of the dentist, keeping the mouth open, teeth being cleaned by a dentist or nurse, instruments put into the mouth, and suction used in the mouth. The treatment of dental caries (TC) subscale included following items: drilling, hearing the sound of drilling, local anesthesia, pain, and being unable to breathe. The response alternative of 6 was replaced with the mean of other items for that respondent, as previous replacement methods have been difficult to interpret [28]. For seven items, there was one missing value for each, and of these, six values were imputed; for one item, two cases with missing values were observed, and the missing values were imputed; and for one item (teeth cleaned), four missing values were observed, and one of them was imputed all with mean of other items.

Construct validity was assessed by examining differences in MDAS scores with descriptive statistics and statistical tests between genders, age (here equal to grade), and according to treatment received [29], as these differences have been found in previous studies in children [1,19] and in adults [22]. The latter was carried out by using continuous and categorized DFA variables. Differences between groups were analyzed using chi-square tests for categorized variables and with Mann–Whitney and Jonckheere–Terpstra tests for continuous variables that were not normally distributed. Analyses were also conducted separately for different grades and genders. Criterion validity was assessed with Spearman rank correlation coefficients between MDAS and its subscales against CFSS-DS-M and its subscales and against the single question. The reliability of MDAS in terms of internal consistency was evaluated using Cronbach’s alpha. The statistical significance was considered at a *p*-value of 0.05. Statistical analyses were conducted using IBM SPSS Statistics version 30.3 (SPSS Inc., Chicago, IL, USA).

## 3. Results

A total of 57 of the third graders and 69 of the sixth graders were present at the school and participated in the study, leading to 85.1% and 93.2% response rates, respectively. The mean age for the third graders was 9.4 years (SD 0.49) and 12.3 years (SD 0.44) for the sixth graders. The distribution of the participants according to grade, gender, treatment experiences, and DFA measured using both scales is presented in Table 1. The differences in DFA between grades were not statistically significant. Children in sixth grade reported more often having experienced extractions, local anesthesia, and orthodontics than third graders. The means (SD) for the single DFA questions were 1.7 (0.9) and 1.7 (0.94) for boys and girls, respectively. Girls reported more often experiencing orthodontic treatment than boys (66% vs. 34%, *p* = 0.021), but for other treatments, there were no gender differences.

When looking at gender differences in DFA according to grade, girls in third grade tended to have lower mean ADA (3.4, SD = 1.44) than boys (4.5, SD = 2.21, *p* = 0.051). In sixth graders, girls tended to have higher mean TRA (8.4, SD = 3.17) than boys (6.9, SD = 2.61, *p* = 0.067). These differences according to gender across grades indicate construct validity for MDAS despite *p*-values being below the set level for statistical significance.

There were only a few statistically significant differences between those reporting treatment experiences and those not reporting (Table 2). Children who reported orthodontic treatment reported lower ADA than children not reporting experience of orthodontic treatment. On the other hand, children who reported experience of extractions or local anesthesia reported higher TC than those who did not report having experience of these procedures. In sixth graders, similar differences were observed for local anesthesia and orthodontics. The mean (SD) for ADA was 3.2 (4.77) for those who reported experience of orthodontic treatment and 3.9 (1.73) for those not reporting (*p* = 0.009, Mann–Whitney test). The mean (SD) for TC was 11.3 (4.69) for those reporting experience with local anesthesia and 9.1 (4.13) for those not reporting (*p* = 0.029, Mann–Whitney test). In third grade, the means (SD) for CFSS-DS-M sum (23.1 (9.83)) and TC (13.5 (5.00)) were higher for those reporting experience of local anesthesia: 17.5 (9.226) and 9.6 (5.05), respectively. The results indicate construct validity for MDAS according to the treatment received but in a different subscale than CFSS-D-M.

Correlations between MDAS and CFSS-DS-M and their subscales for all participants and according to grade are presented in Table 3 and according to gender in Table 4. The correlations ranged from high to moderate for most groups, but the correlation between ADA and AD was weaker for boys. The correlations between the single question of DFA and MDAS sum, ADA, and TRA were 0.564, 0.584, and 0.442, respectively (all *p* < 0.001). The correlation coefficients indicate good criterion validity for MDAS and its subscales.

Cronbach alphas were 0.841, 0.708, and 0.778 for MDAS total, ADA, and TRA, respectively. Corresponding alphas for third graders were 0.799, 0.708, and 0.709 and for sixth graders 0.875, 0.832, and 0.837, respectively. The results suggest good reliability for the MDAS.

## 4. Discussion

The Finnish version of the Modified Dental Anxiety Scale (MDAS) showed good reliability and good criterion validity when compared to the Finnish modified Child Fear Survey Schedule—Dental Subscale (CFSS-DS-M) total scale and its subscales as well as for the single dental fear and anxiety (DFA) question. The construct validity was also acceptable, as MDAS found differences between genders and treatment received.

Overall, the criterion validity of the MDAS was supported by moderate to strong correlations with the CFSS-DS-M across the total sample. Correlations were stronger in younger children and in girls. This may suggest that DFA is reported more consistently among younger children and among girls, whereas boys and older children may express dental anxiety in more variable or fragmented ways. These findings may reflect developmental and gender-related differences in how DFA is experienced and reported and highlight the importance of considering age and gender when interpreting DFA measures in children. The low number of missing values supports the use of MDAS already for children from age 9 years, as children did not seem to experience more cognitive or linguistic problems in responding to the MDAS compared to the CFSS-DS-M. Thus, the questions and instructions for children seemed to be simple enough, and the wording was clear and unambiguous, as suggested by Borgers et al. [23]. Like in a study in 8- to 15-year-old children, the number of missing values did not vary according to age, further supporting the validity [13].

The construct validity of the MDAS was better for the subscales of MDAS than for the CFSS-DS-M. The differences in the mean MDAS, ADA, and TRA scores between genders were quite large, i.e., over one point, though the statistical significance was below the set level. The ability of CFSS-DS-M to identify differences according to gender was not good either. In Finland, gender differences in DFA were not observed in 9- or 12-year-olds but only in 15-year-olds when using CFSS-DS-M [30] and in 11- to 12-year-olds when using the single question [31]. For gender or age, which is equivalent to grade in our study, differences have not been consistent in previous studies [1,32].

The fact that those who had experienced orthodontic treatment had lower ADA might be because they had had more visits and more treatment procedures, which are common in orthodontics. Thus, orthodontic treatment might have worked as desensitization, which has been useful in managing DFA [33]. As girls had experienced orthodontic treatment more often, this might also explain part of the gender differences. On the other hand, the TC of CFSS-DS-M performed better in finding differences according to treatment procedures, which might be due to more items, especially including an item on pain. On the other hand, many CFSS-DS-M AD items (such as suction or instruments used in the mouth) are also related to procedures in dental chairs. In contrast, MDAS AD items measure DFA feelings before entering the operatory. Thus, MDAS might better capture the DFA of those children whose fear originates from sources other than their own direct experiences. Anticipatory elements of DFA have also been found theoretically relevant [14]. Children who lack dental visit experience or have experience with caries treatment were also at increased risk of DFA, according to recent meta-analyses [2,34]. In a previous Finnish study, family members’ DFA was also an equal or even more important predictor of children’s DFA [30].

The strength of this study was a sample that represents children from various socio-economic groups. This is because, in Finland, practically all children attend comprehensive schools that are publicly funded and free of charge. Additionally, children are assigned to the schools, and thus, parents are practically not allowed to choose the school they want. The use of paper forms in the presence of teachers decreased systematic bias. Another strength was that the response rates were high because the questionnaires were applied at school.

Our study also has limitations. Though we performed a sample size calculation, the sample size might have been too small to detect differences. For example, the differences in MDAS dimensions were only marginally significant, though a point difference in one dimension can be considered rather large. The use of a convenience sample also limits the generalization of the findings. The use of self-report measures is a potential source of bias. DFA measures might have had social desirability bias, as the presence of schoolmates can influence their answers [23]. However, for MDAS and CFSS-D-M, the bias is likely to work in the same direction. Another source of bias may be that it is socially more acceptable for girls than boys to express DFA. The questions on oral healthcare treatment procedures have a potential recall bias. Children might remember better the procedures in which they had negative experiences. Additionally, we did not use measures, such as family environment, that assess other origins of DFA besides treatment experiences. Dental anxiety has other origins besides direct experiences [15], and assessing these would have given a possibility to assess construct validity against them. Thus, further studies with larger and more diverse samples and including other variables related to child DFA are needed to test the use of MDAS in child populations.

Measuring DFA with reliable and valid scales is important, as oral health personnel do not seem to be able to assess DFA without them [35,36]. Parents and children are also not able to assess each other’s DFA [25,26]. When assessing the parental and child DFA and their association, valid and reliable measures should be used [24]. Using a similar assessment method may increase the comparability. MDAS also seems to better capture anticipatory dental anxiety and possibly identify those at risk to avoid further visits, as also suggested for adults [14].

Based on our results, we conclude that the Finnish version of the Modified Dental Anxiety Scale (MDAS) showed good reliability, good criterion validity, and acceptable construct validity among children aged 9 to 12 years. Thus, it can be used to measure the DFA of children.

## Figures and Tables

**Table 1:** Distribution of the participants according to grade, gender, treatment experiences, and dental anxiety.

	Third Graders n = 57	Sixth Graders n = 69	*p*-Value	All
Gender n (%)				
Boy	31 (54.4)	31 (45.6)	0.327	62 (49.6)
Girl	26 (45.6)	37 (54.4)		63 (50.4)
Treatment experiences, yes n (%)				
Fillings	17 (29.8)	30 (44.8)	0.087 *	47 (37.9)
Extraction	9 (16.4)	22 (32.8)	0.038 *	31 (25.4)
Orthodontics	10 (18.2)	31 (47.0)	<0.001 *	41 (33.9)
Local anesthesia	17 (30.4)	40 (59.7)	0.001 *	57 (46.3)
MDAS ^1^, mean (SD)				
Total	12.3 (4.60)	11.3 (4.50)	0.153 ^§^	11.8 (4.56)
ADA ^2^	4.0 (1.95)	3.6 (1.90)	0.110 ^§^	3.7 (1.92)
TRA ^3^	8.4 (3.16)	7.7 (3.00)	0.168 ^§^	8.0 (3.08)
CFSS-DS-M ^4^, mean (SD)				
Total	16.2 (7.05)	16.0 (6.33)	0.609 ^§^	16.1 (6.63)
AD ^5^	7.3 (3.60)	6.7 (2.98)	0.306 ^§^	7.0 (3.27)
TC ^6^ mean (SD)	9.0 (4.34)	9.4 (4.15)	0.992 ^§^	9.2 (4.23)
How afraid of dental treatment in general, mean (SD)	1.8 (1.03)	1.6 (0.90)	0.210 ^§^	1.7 (0.96)

^1^ MDAS = Modified Dental Anxiety Scale; ^2^ ADA = anticipatory dental anxiety subscale of MDAS; ^3^ TRA = treatment-related dental anxiety subscale of MDAS; ^4^ CFSS-DS-M = Child Fear Survey Schedule—Dental Subscale, modified; ^5^ AD = attending dentists subscale of CFSS-DS-M; ^6^ TC = treatment of dental caries subscale of CFSS-DS-M. *p*-values * for chi-square test and ^§^ for Mann–Whitney test.

**Table 2:** Mean (SD) dental anxiety levels according to treatment procedures for all participants.

	Filling		Extraction		Local Anesthesia		Orthodontics	
	Yes (n = 43)	No (n = 68)	Yes (n = 27)	No (n = 82)	Yes (n = 53)	No (n = 57)	Yes (n = 39)	No (n = 69)
MDAS ^1^	11.4 (3.89)	12.0 (4.95)	11.4 (4.10)	12.1 (4.70)	11.6 (4.20)	12.1 (4.87)	11.7 (4.74)	12.0 (4.48)
ADA ^2^	3.7 (1.73)	3.6 (2.06)	3.6 (1.95)	3.8 (1.95)	3.8 (2.00)	3.7 (1.90)	3.4 (2.13)	4.0 (1.83) **
TRA ^3^	7.7 (2.76)	8.3 (3.26)	7.8 (2.65)	8.3 (3.18)	7.8 (2.72)	8.4 (3.32)	8.3 (2.92)	8.1 (3.12)
CFSS-DS-M ^4^	18.4 (7.77)	17.9 (8.72)	19.4 (7.43)	17.8 (8.66)	19.5 (8.50)	16.9 (8.14)	18.5 (7.93)	18.1 (8.71)
AD ^5^	7.2 (3.53)	7.6 (4.15)	7.3 (3.91)	7.5 (3.97)	7.5 (4.22)	7.4 (3.69)	7.1 (3.65)	7.7 (4.12)
TC ^6^	11.2 (4.61)	10.2 (5.01)	12.1 (4.34)	10.2 (4.98) *	12.0 (4.83)	9.4 (4.65) ***	11.3 (4.89)	10.4 (4.90)

^1^ MDAS = Modified Dental Anxiety Scale total; ^2^ ADA = anticipatory dental anxiety subscale of MDAS; ^3^ TRA = treatment-related dental anxiety subscale of MDAS; ^4^ CFSS-DS-M = Child Fear Survey Schedule—Dental Subscale, modified total; ^5^ AD = attending dentists subscale of CFSS-D-M; ^6^ TC = treatment of dental caries subscale of CFSS-DS-M. * *p*-value for Mann–Whitney test < 0.05; ** *p*-value for Mann–Whitney test < 0.01; *** *p*-value for Mann–Whitney test < 0.001.

**Table 3:** Spearman rank correlation coefficients between MDAS and CFSS-DS-M and their subscales according to grade. All *p*-values < 0.001.

	3rd Graders			6th Graders			All		
	CFSS-DS-M ^4^	AD ^5^	TC ^6^	CFSS-DS-M ^4^	AD ^5^	TC ^6^	CFSS-DS-M ^4^	AD ^5^	TC ^6^
MDAS ^1^	0.784	0.794	0.772	0.581	0.559	0.563	0.674	0.626	0.655
ADA ^2^	0.735	0.675	0.684	0.513	0.538	0.478	0.602	0.594	0.566
TRA ^3^	0.654	0.610	0.649	0.565	0.516	0.551	0.605	0.566	0.583

^1^ MDAS = Modified Dental Anxiety Scale total; ^2^ ADA = anticipatory dental anxiety subscale of MDAS; ^3^ TRA = treatment-related dental anxiety subscale of MDAS; ^4^ CFSS-DS-M = Child Fear Survey Schedule—Dental Subscale, modified, total; ^5^ AD = attending dentists subscale of CFSS-DS-M; ^6^ TC = treatment of dental caries subscale of CFSS-DS-M.

**Table 4:** Spearman rank correlation coefficients between MDAS and CFSS-D-M and their subscales according to gender. All *p*-values < 0.001 except * *p* = 0.002.

	Boys			Girls		
	CFSS-DS-M ^4^	AD ^5^	TC ^6^	CFSS-DS-M ^4^	AD ^5^	TC ^6^
MDAS ^1^	0.558	0.499	0.569	0.766	0.727	0.733
ADA ^2^	0.443	0.395 *	0.432	0.746	0.758	0.695
TRA ^3^	0.536	0.503	0.541	0.660	0.627	0.621

^1^ MDAS = Modified Dental Anxiety Scale total; ^2^ ADA = anticipatory dental anxiety subscale of MDAS; ^3^ TRA = treatment-related dental anxiety subscale of MDAS; ^4^ CFSS-DS-M = Child Fear Survey Schedule—Dental Subscale, modified total; ^5^ AD = attending dentists subscale of CFSS-DS-M; ^6^ TC = treatment of dental caries subscale of CFSS-DS-M.

## Data Availability

Data are not available due to restrictions related to privacy and ethical issues. The datasets presented in this article are not readily available because of restrictions based on EU GDPR and local legislation. Requests to access the datasets should be directed to S.L.

## Abbreviations

The following abbreviations are used in this manuscript:

DFA	Dental fear and anxiety
CFSS-DS	Child Fear Survey Schedule—Dental Subscale
DAS	Dental Anxiety Scale
MDAS	Modified Dental Anxiety Scale
S-DAI	Short version of the Dental Anxiety Inventory
SFP-R	Smiley Faces Program—Revised
DFS	Dental Fear Survey
MCDAS	Modified Child Dental Anxiety Scale
ADA	Anticipatory dental anxiety subscale of MDAS
TRA	Treatment-related dental anxiety subscale of MDAS
CFSS-DS-M	Child Fear Survey Schedule—Dental Subscale—Modified
AD	Attending dentists subscale of CFSS-DS-M
TC	Treatment of dental caries subscale of CFSS-DS-M
SD	Standard deviation
